# The Touting System Amongst Dentists

**Published:** 1850-10

**Authors:** 


### ARTICLE IV.

### *The Touting System amongst Dentists.*

Every one at all conversant with Goldsmith's Tales, must remember the story of the celebrated quack doctor and his son, who were in the habit of traveling through the country entrapping the unwary, and plundering the simple and credulous. The son generally preceded the father by a few days, and mounted on a scaffold in the market place, announced by sound of trumpet and beat of drum, his parent's advent, pronouncing a high eulogium upon his learning, his skill, his miraculous cures, and his still more miraculous cheapness. The doctor on his arrival found the harvest ripe for the sickle and sheared the simpletons accordingly, leaving them to find out the cures and to calculate their losses at their leisure, while he proceeded on his way to fleece the next batch. Whether Goldsmith's doctor was the prototype of the touting system that prevails so extensively in the present day, we cannot take upon ourselves to say, but this much is certain, that a practice discreditable and undignified in itself was formerly confined to low pettifoggery, old Baily lawyers, linen drapers, tavern keepers and others of that class, has of late been introduced into the medical profession and its offshoots. And we regret to say, has been adopted by men who have some pretensions to professional skill and medical science, and who do not hesitate to tout for practice and patronage from all who have any claim to respectability.

With pure members of the medical profession, it is not our province to deal, "the Times of Medical Literature," "the Lancet" has repeatedly pointed severe strictures against the wrong-doers, and called them to account for breaches of professional etiquette. To the editor of that journal, therefore, we consign the touting members of the medical profession, feeling assured that the honor and character of the body he represents will be perfectly safe in his hands. When we find the higher branches of medicine and members of the legal profession resorting to such practices, we need not be much surprised that among the offshoots and collateral branches of surgery, the system has increased to a frightful extent. Among the members of a profession where competition is severe, the temptation to resort to any means by which practice may be obtained is undoubtedly great, and they are too happy to be enabled to point to men of higher standing and pretensions, imitate their bad example and shelter themselves under the plea that "others have done so before them." Among the varied phases and aspects under which professional competition exhibits itself, touting is probably that which is most likely to find imitators and disciples. The success of one adventurer induces hundreds to follow in the same path, the infection spreads like an epidemic, and unless checked by wholesome ridicule and exposure, will probably obtain general sanction and recognition.

Among the various branch practitioners, dentists may be said to be prone beyond all others to carry out the touting system. It is a department of surgery in which large numbers are to be found peculiarly adapted for such practices ; we may instance the large number who have commenced as dentists without the remotest qualifications, and who resort to this extraneous mode of procuring practice; there are also those who are induced to follow the example of their neighbors, those who do it from choice, those who resort to it for want of knowing better, and those compelled by necessity, while all evince a want of proper respect for themselves and the profession. Men half educated both in medical and dental science are seldom very scrupulous as to the means by which a practice may be obtained. Having no confidence in themselves, they rely upon the facility with which the credulous public may be led to adopt the exaggerated estimate of their skill which their "trumpeters" put forth and reap the fruits of a system which is virtually one of fraud and deception.

The methods resorted to by your dental touter are in many instances curious and extraordinary, and depend upon the resources, fancies and ingenuity of the practitioner. Some direct their energies to a display of their talents by means of fancy door and window case exhibitions, of the style of work performed within, while a boy, all over buttons, thrusts lists of prices into the hands of every passer by, stating the tariff of charges, from a set of artificial teeth down to a single grinder, with N. B. all consultations free. Others of about the same grade of respectability exhibit large placards and posters on traveling vans, announcing an important discovery in artificial teeth, and stopping with the instruction, no connection with any other person of the same name, practicing as a dentist. Others inundate a whole district with circular letters containing a list of peculiarly tempting prices, extraordinary discoveries, wonderful cures and apocryphal testimonials, and allowances for a series of operations, and winding up occasionally with some doggerel verses, on some compound for filling decayed teeth.

These departments of touting are, on the whole, inoffensive, and are adopted by those whose means are limited, and who seldom venture beyond the mere exhibition distinguishing their trade. But there is another method resorted to, by means of advertising, and in this way large sums of money are annually expended; here we find all classes of touters setting forth their prices and pretensions to patronage, and really some of these advertisements contain very interesting and amusing specimens of laudatory literature. We have Teeth, Teeth! Pretty Teeth! Beautiful Teeth! a New Discovery; every advertisement containing some wonderful secret, of course known only to the advertiser. The simple public is led to believe that it is as easy to have your mouth filled with ready made teeth, as it is to go to Moses & Sons, the cheap tailors, and be fitted with a ready made pair of breeches. You have only "to open your mouth and *pocket*, shut your eyes" and you will find yourself the *fortunate* purchaser of some second hand misfit, neither over clean, ornamental, nor very useful artificial grinders.

This department of touting is always attended with great expense and may be looked upon as a dental gambling speculation. It requires ability and judgment to carry on the system, to devise the best means of circulation, and of framing catching advertisements, and above all, it requires a certain amount of capital. Newspaper proprietors having of late years entertained *well* grounded doubts of the stability and pecuniary resources of this class of professors of dentistry, and demand prompt payment. Young and inexperienced touters continue for a time advertising, and all at once disappear from the stage, the means cannot be found, and as no credit is given they soon discover that they cannot make head against speculative capitalists in advertising for patronage. Those who have the necessary amount of funds to persevere, succeed no doubt, to some extent, as there are always a certain class of persons on the *qui vive* for cheap, dirty and deleterious artificial teeth and dental operations. Nevertheless, few of them realize even a competence, and being dependent on the large outlay they are compelled to resort to.

We have hitherto referred only to the struggling class who maintain themselves by direct self laudation, but there are many other curious specimens of those who follow the *trade* of dental surgery and whose ingenious but not very respectable efforts to obtain notoriety and practice, must excite the risible faculties of all who are favored with a peep behind the curtain. Among the many varieties whom we should sketch for the amusement of our readers, if time permitted, the most amusing and instructive are the touters of a different standing, comprising men, many of whom, one would suppose too well conditioned at the commencement of their professional career, judging from their selfestimation and importance, to have recourse to a system of touting. As we before observed in other articles, England has long been the favored land of professional humbug; there it flourishes in full luxuriance as regards dentistry, and one of the most ludicrous and occasionally successful is that of your gastronomic touter. John Bull has from time immemorial been proverbial for his love of the good things of this life; mention the word dinner, and immediately visions of huge haunches of venison, turtle and champagne start up and float in his imagination till the day and hour arrive, neither time nor distance is allowed to interfere, other occupations are put aside and miles of railway traversed if necessary, to enable him to ensconce himself at the appointed hour under the "mahogany" of your real dental gastronomic practitioner.

The professors of this kind of touting are men either commencing a professional career, or who have purchased a practice or position, and who are of course desirous of retaining in their service the corps of walking gastronomic advertisers. The majority may be said to be self-educated, either in medical or dental science, and the only chance they have of securing a practice, is in giving extravagant dinners to M. D's. and surgeons in embryo, specialists endeavoring to form a connection through the medium of *name only* of some eminent practitioner, while perhaps a medical knight or baronet will occasionally *lend* his countenance to the entertainment, as the patron of the successor, the hunter of fashionable patients, and the lion of the feast.

In these cases, the touter calculates with arithmetical precision the expenses of his entertainment, the profit per contra to be derived from each of his guests, and the value of each, individually, as a walking pair of bellows, in blowing forth his professional fame. To do John Bull justice, he is seldom ungrateful. You have only to ring him through the nose, ply him with champagne and venison, and he is your fashionable touter's firm friend—the good natured, stupid animal can be made to say or to do any thing for the prospect of a repetition of the feast. In fact, a close account is kept by your dinner-giving touter, of the value of each individual guest to him, in returning the *quid pro quo*, in the shape of patients and recommendations. If the return be not adequate to the value expended, the M. D's. and specialists may rest assured that no repetition of the touter's *disinterested* hospitality will be experienced, and the intimacy will speedily thaw down to a how-d'ye-do and the cold shoulder. Norton's broadest farce furnishes nothing more ludicrous than the exaggerated boastings of one of this class when a brother professional chances to come in contact with him. Your real dinner touter will have you to believe that he has discovered some secret remedy for filling teeth, some curious instrument invented and used only by himself, his instrument maker having strict injunctions not to part with the patterns; that the same was examined and approved of by Dr. Boobey and Mr. Alexander Twaddle, when dining with him the other day. The conversation is occasionally flavored by throwing in a little fashionable seasoning. Lady A., the Earl of B., &c., are confidentially named as persons on whom he has tried his new inventions, by the recommendation of his gastronomic friends; and if you do not leave him impressed with a high opinion of his abilities and his professional standing, it will not be owing to any want of industry or zeal on his part in puffing them off.

This system of touting for practice is said to be very expensive and very unsatisfactory. Like the Spanish blood-hounds employed in hunting down the Indians, who required to be occasionally regaled with a taste of the game to keep the scent alive, the friend of the dinner-giving touter must have his stomach kept well filled if he is expected to run down the game. Besides, many swallow the bait, but will not allow themselves to be hooked. They will partake of the touter's hospitality, appreciate the dinner, speak well and drink deep of the wine, eulogize the kindness of the host during the process of the banquet, but forget the main object for which they have been crammed and feasted, viz. to impress their friends and acquaintances with a due sense of his professional ability. These thankless and unprofitable gourmands, although accepting and enjoying the feast, frequently most ungraciously consult another practitioner in matters in which professional skill is required, and laugh in their sleeves at the overweening vanity and egregious folly of your professional dental gastronomic dinner-touter.

Notwithstanding all its drawbacks, there is little doubt that the touting dinner system answers the purposes of some practitioners, and brings around them a clique of men of uncertain professional character, who keep good living and frequent entertainments in view at the expense of reputation and discretion. The patron of the protegé become sometimes mixed up in the percentage system, (of which we shall have a few words to say on a future occasion,) and the result of which ultimately is exposure and disgrace to both.

We believe that in this, as in many other instances, the calling of professional and public attention to a discreditable system will go a great way towards correcting it—that the bringing the force of public opinion to bear upon professional abuses, is one of the most efficient means of putting them down, and we feel satisfied that the only true way of ensuring the respect of the profession and the public at large is to begin by exhibiting a proper respect for ourselves, to abandon all the artifices and tricks of quackery and pretence, and by so doing, we shall best raise the dental art to the high and honorable position which it is fairly entitled to occupy among the collateral branches of medicine and surgery.

